# Development of Europium-Sensitized Fluorescence-Based Method for Sensitive Detection of Oxytetracycline in Citrus Tissues

**DOI:** 10.3390/antibiotics10020224

**Published:** 2021-02-23

**Authors:** Faraj Hijaz, Yasser Nehela, Pedro Gonzalez-Blanco, Nabil Killiny

**Affiliations:** 1 Department of Plant Pathology, Citrus Research and Education Center, IFAS, University of Florida, 700 Experiment Station Road, Lake Alfred, FL 33850, USA; fhijaz@ufl.edu (F.H.); yasser.nehela@ufl.edu (Y.N.); pcgo@ufl.edu (P.G.-B.); 2 Department of Agricultural Botany, Faculty of Agriculture, Tanta University, Tanta 31512, Egypt

**Keywords:** Huanglongbing, oxytetracycline, OTC, europium, 4-epi-OTC, antibiotic, citrus

## Abstract

Antimicrobial compounds have been successfully used to control many plant and animal diseases. Recently, oxytetracycline (OTC) and streptomycin have been approved for the treatment of Huanglongbing in citrus. Since the application of OTC is under strict regulations, several methods have been developed to determine and monitor its levels in the environment including high-performance liquid chromatography, ELISA, colorimetric, and fluorometric assays. In this study, we developed a fluorometric method for the determination of OTC in plant tissues based on its complexation with europium. Our preliminary trials showed that phenols and flavonoids interfere with the europium assay by reacting with the sensitizing reagent, cetyltrimethylammonium chloride. Consequently, we used the 60 mg hydrophilic–lipophilic balanced (HLB) cartridges to purify the OTC from the plant matrix. The recovery of OTC from spiked leaf samples was 75 ± 7.6%. Using the 500 mg HLB, we were able to detect 0.3 ppm OTC in the final sample extract, which corresponds to 3 µg g^−1^ fresh weight (FWT). The developed method was successfully used to measure the level of OTC in leaves obtained from trunk-injected trees. The results obtained by the europium method were similar to those obtained using the ELISA assay. We also tested the cross-reactivity of OTC metabolites with the europium method. The 4-*epi*-OTC showed a high cross-reactivity (50.0 ± 3.6%) with europium assay, whereas α-*apo*-OTC and β-*apo*-OTC showed small cross-reactivity. We showed that the europium-sensitized fluorescence-based method can be successfully used to assess OTC in citrus plant tissues after a cleanup step. Our results showed that this method was sensitive, reproducible, and can be used to analyze many samples simultaneously.

## 1. Introduction 

Antimicrobial compounds including oxytetracycline (OTC) and streptomycin have been effectively used to control many plant diseases since the middle of the 20th century [1]. For example, OTC has been used for the control of the yellow diseases in coconut palm and elm trees, which is caused by phytoplasmas [2]. OTC has also been registered for the control of spot and fire blight diseases on beaches [2]. OTC has also been used for the control of *Xanthomonas* spp. and *Pseudomonas* spp. on different vegetables [2].

OTC and streptomycin were also approved for the control of Huanglongbing in citrus groves [3]. This decision came after the substantial losses in the citrus industry that were caused by Huanglongbing. The notion of using antibacterial agents for the control of Huanglongbing was proposed in the 1970s after it was suggested that it was caused by a bacterial pathogen [4]. However, the use of oxytetracycline was stopped because it has to be applied frequently, which could result in phytotoxicity [5]. Huanglongbing is believed to be caused by the bacterium, *Candidatus* Liberibacter asiaticus (*C*Las), which is transmitted by the Asian citrus psyllid, *Diaphorina citri*. The Asian citrus psyllid transmits the Ca. L. asiaticus pathogen while feeding on citrus phloem sap. Previous studies showed that the use of antibiotics including ampicillin, tetracycline, penicillin, and rifampicin was effective against the Ca. L. asiaticus [5].

Trunk injection of 2 g of OTC into 5-year-old ‘Hamlin’ sweet orange trees resulted in high levels of OTC (>850 µg/kg) in leaf and root and reduced the *C*Las titer by more than 99% 28 days post-injection [6]. The injection of OTC improved fruit yield and slightly reduced juice acidity, whereas total sugars and juice content were not affected [6]. A trace amount of OTC (202 µg/kg) was detected in fruits nine months after treatment [6]. Recently, we showed that trunk injection of OTC was more efficient than foliar application [7]. The *C*Las titer was significantly reduced in oxytetracycline-injected trees thirty days after treatment, whereas it was not affected by foliar application. The addition of various adjuvants to the OTC solution did not enhance its uptake by citrus leaves [7].

OTC is also used in animals to control several pathogenic bacteria [8]. OTC could accumulate in meats because it requires a long time to be eliminated from the animal body [8]. The level of OTC and its main metabolite, 4-*epi*-OTC, in foods are under strict regulation because their accumulation can result in bacterial resistance [8]. Several methods have been developed to determine the level of OTC in animals and foods. These methods included high-performance liquid chromatography with UV-visible and mass spectrometry detection [8]. Although these methods can be used to measure OTC in various matrixes, they need expensive instruments and cannot be used to analyze a large number of samples at the same time. In addition, several colorimetric methods using polyvalent cations such as cupric, iron, and zirconium were used for chelation OTC [9]. These colorimetric methods are quick and simple. However, these methods are not sensitive and cannot be used to determine the level of OTC in complex matrixes without cleanup steps.

In general, the enzyme-linked immunosorbent assay (ELISA) is the most desired method for the detection of OTC and other antibiotics because it is sensitive, simple, quick, and can be used to analyze a large number of samples simultaneously. Recently, we used the ELISA assay to study the uptake of OTC in citrus plants [10]. Using the ACCEL ELISA method, we were able to detect OTC in the leaves, root, phloem, and xylem [10]. The ACCEL ELISA assay is very sensitive but has a narrow linear range (1.56–50 ng mL^−1^). Although the OTC ELISA assay is fast and convenient, it is very expensive [11].

**Figure 1:**
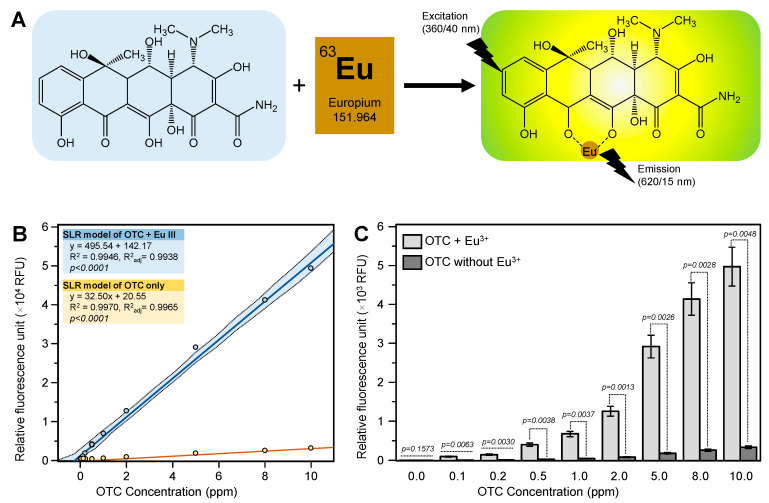
Complexation of europium with oxytetracycline (OTC). (**A**) Formation of the fluorescent 1:1 β-diketone:Eu(III) complex via the BCD (second to fourth) rings hydroxyl groups of OTC. The OTC absorbs. The OTC attached to the europium ion strongly absorbs at 388 nm and passes the absorbed energy to europium, which emits it as a narrow and intense peak at 615 nm. (**B**) OTC standard with and without europium. (**C**) Relative fluorescence intensity of OTC with and without europium. Values with *p*-values < 0.05 are significantly different using two-tailed student *t*-test. Assay was conducted using 100 µL standard, 300 µL Tris buffer (0.1 M, pH 8.5), 40 µL ethylenediaminetetraacetic acid (EDTA) (0.00025 M), 40 µL cetyltrimethylammonium chloride (CTAC), and with or without 20 µL europium (0.00125 M).

Early studies showed that complexation between europium and tetracyclines in alkaline conditions results in luminescence emission, which can be significantly enhanced by the addition of surfactants such as cetylpyridinium chloride (Figure 1A) [12]. The tetracycline ligands attached to the europium ion strongly absorb at 388 nm and pass the absorbed energy to europium, which emits it as a narrow and intense peak at 615 nm (Figure 1A) [13]. This method has been successfully used to measure the tetracycline levels in different biological samples including milk, calf serum, and chicken muscle, blood serum, urine, and gingival crevice fluid [13,14]. However, this method has not been used for the detection of OTC in plant tissues. The objective of this study was to develop a fluorometric method for the determination of OTC in citrus leaves based on its complexation with europium in alkaline solutions. 

## 2. Results

### 2.1. Plant Matrix Interferes the Europium-Sensitized Fluorescence Intensity

The fluorescence intensity of the OTC standard was significantly enhanced (about 15-fold) by the addition of europium (Figure 1B,C). This result indicated that the europium method was a sensitive method for the detection of OTC. In our preliminary work, we tried to analyze spiked citrus leaf samples without any further cleanup. Unfortunately, the spiked samples showed very low fluorescent intensity compared with the pure OTC standard. The pH of the sample (8.5 ± 0.1) after being mixed with the assay’s reagents was similar to that of the standard (8.5 ± 0.1), indicating that the pH was not the problem. We also noticed a yellow color when the sample was mixed with the Tris buffer, indicating a presence of citrus plant metabolites in the sample extract. 

### 2.2. Phenols and Flavonoids Are Associated with the Interference of Europium Assay 

**Figure 2:**
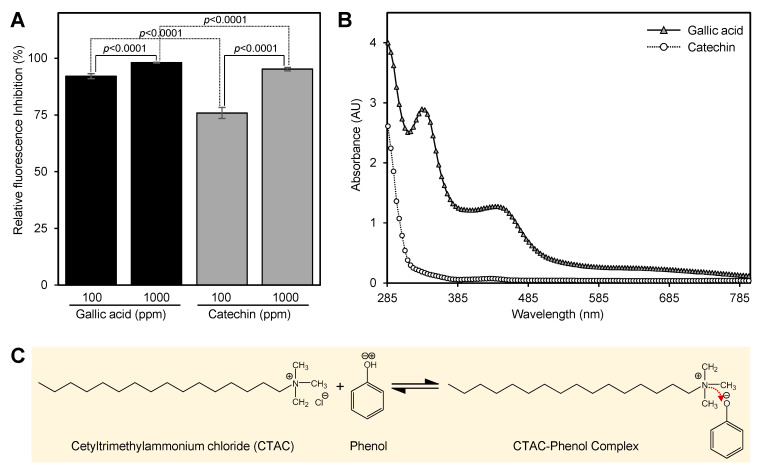
Interference of phenols and flavonoids with the europium method. (**A**) Relative fluorescence inhibition (%) of gallic acid and catechin when present in the final assay at 100 and 1000 ppm. (**B**) The UV-visible spectra of gallic acid and catechin with CTAC in tris buffer (pH 8.5). (**C**) Reaction scheme between CTAC and phenol. Values with *p*-values < 0.05 are significantly different using two-tailed Student *t*-test.

Consequently, we decided to test the interference of phenols and flavonoids with europium assay. Gallic acid and catechin were chosen as a representative for phenols and flavonoids, respectively. Gallic acid showed 91.1 ± 1.1 and 98.1 ± 0.2% inhibition when present at 100 and 1000 ppm in the final assay’s mixture, respectively (Figure 2A). Catechin showed lower fluorescence inhibition compared with gallic acid (Figure 2A); it showed 75.8 ± 2.4 and 95.2 ± 0.8% inhibition when present at 100 and 1000 ppm in the final assay’s mixture, respectively (Figure 2A). Further investigation showed that gallic acid forms a yellow complex with cetyltrimethylammonium chloride (CTAC) at high pH (Figure 2B). This complex showed two shoulder peaks (335 and 445 nm) (Figure 2B). The absorption intensity of the catechin–CTAC complex was also lower than that of the gallic acid–CTAC spectrum (Figure 2B). The reaction of CTAC with phenol is considered an acid–base reaction (Figure 2C).

### 2.3. Surfactant Addition Increases the Fluorescence Intensity

**Figure 3:**
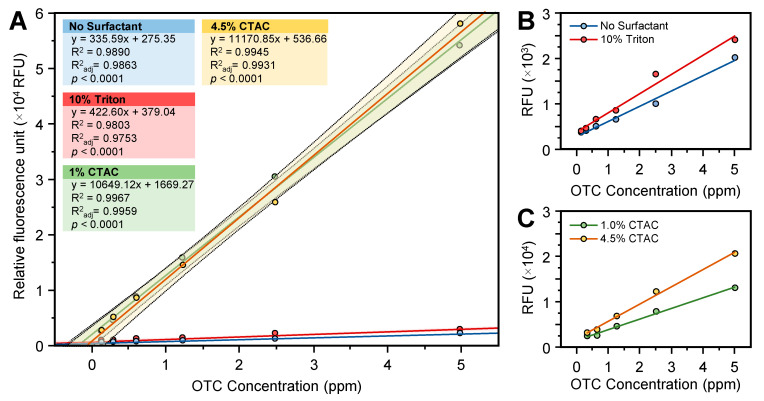
Effect of surfactant on the fluorescence intensity of the OTC-Eu(III) complex. (**A**) Fluorescence intensity of OTC standard with 1% CTCA, 4.5% CTCA, 10% Triton X-100, and without any surfactant. (**B**) Fluorescence intensity of OTC standard with 10% Triton X-100 and without any surfactant. (**C**) Fluorescence intensity of OTC standard (prepared in sample matrix and cleaned using 60 mg hydrophilic–lipophilic balanced (HLB) cartridge) with 1 and 4.5% CTCA. Assay was conducted using 100 µL standard, 300 µL Tris buffer (0.1 M, pH 8.5), 40 µL EDTA (0.00025 M), 20 µL europium (0.00125 M), and 40 µL surfactant.

Our preliminary work showed that OTC has very low fluorescence intensity in the absence of europium (Figure 1B,C). Likewise, the OTC-Eu(III) complex showed very low fluorescence intensity without the addition of any surfactant (Figure 3A). The addition of 40 µL of 10% of Triton X-100 slightly increased the fluorescence of the OTC-Eu(III) complex (Figure 3A,B). On the other hand, the fluorescence intensity of the OTC-Eu(III) complex was significantly increased after the addition of 40 µL of 1% CTAC surfactant (Figure 3A). The increase of CTAC concentration from 1% to 4.5% did not affect the fluorescence intensity of the OTC standard (Figure 3A). However, the fluorescence intensity of the standard that was prepared using the sample matrix and cleaned using the 60 mg hydrophilic–lipophilic balanced (HLB) cartridge was enhanced by increasing the CTAC concentration (Figure 3C).

### 2.4. Solid-Phase Extraction (SPE)-Based Clean Up Increases the Fluorescence Intensity

In the main study, we used a stronger acid for the extraction of OTC, 1 M HCl containing 2.2% trichloroacetic acid with and without ethylenediaminetetraacetic acid (EDTA). Upon the extraction of OTC from the leaf tissues, the leaf extract was cleaned using solid-phase extraction (SPE). To optimize the SPE, 0.25 mL of the leaf extract (control) was spiked with 25 μL of OTC standard (200 ppm) and applied through the 60 mg HLB cartridge after it was preconditioned with 6 mL of methanol and 6 mL of water.

Initially, the OTC was eluted from the 60 mg HLB cartridge using 1 mL of methanol. However, this gave low fluorescence intensity compared with the eluted standard. Consequently, we tried to clean the OTC before being eluted from the HLB cartridge using a mixture of methanol and water. The spiked sample matrix was washed with 2 mL of 10, 20, 30, 40, 60, and 80% of methanol in water, and then, the OTC was eluted using 1 mL of methanol. These trials showed that OTC starts to elute from the 60 mg HLB cartridge when the percentage of methanol exceeds 20%. We also found that the intensity of OTC was higher when it was eluted using 1 mL of 60% methanol instead of 100% methanol. Therefore, we decided to wash the OTC using 2 mL of 20% methanol and elute it using 1 mL of 60% methanol.

**Figure 4:**
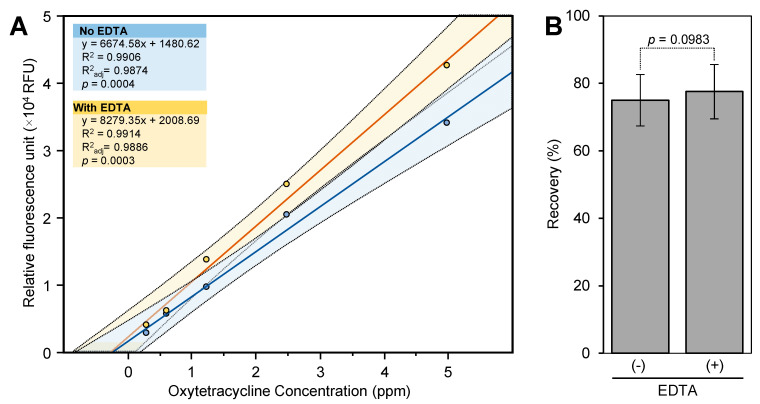
Recovery of OTC from spiked citrus leaf samples using 60 mg HLB cartridge. (**A**) Standard curves of OTC prepared in sample matrix (control samples extracted using 1 M HCl containing 2.2% trichloroacetic acid with and without 0.1% EDTA) and cleaned by eluting 0.25 mL into the 60 mg HLB cartridge. (**B**) Percentage recoveries of OTC from spiked leaf samples extracted using 1 M HCl containing 2.2% trichloroacetic acid with and without 0.1% EDTA. Values with *p*-values < 0.05 are significantly different using two-tailed student *t*-test.

This recovery of OTC from spiked samples was calculated using a standard curve that was prepared in the sample matrix and cleaned up using the 60 mg HLB cartridge (Figure 4A). The recovery of OTC from spiked leaf samples that were extracted with a mixture of HCl and trichloroacetic acid without the addition of EDTA was 75 ± 7.6% (Figure 4B). No OTC was detected in control samples. When the standard curve (constructed using the sample matrix cleaned up using the 60 mg HLB cartridge) was repeated using 4.5% CTCA, the fluorescence intensity of the standard and sample was increased by about 1.5-fold. However, the OTC recovery (88.7 ± 7.5%) was similar to that obtained using 1% CTAC. The addition of EDTA to the extraction solution did not enhance the recovery of OTC (77.5 ± 8.0%) (Figure 4B).

To compare the efficiency of the HLB cartridge with that of the C18 cartridge, the spiked matrix was cleaned using the C18 cartridge under the same condition used for the HLB cartridge. These trials showed that the C18 cartridge was less efficient than the HLB cartridge; the average fluorescence intensity of the spiked matrix eluted from the C18 cartridge was 55.2 ± 7.8% of that eluted from the 60 mg HLB cartridge (n = 5, *p* = value <0.0001). Therefore, we decided to continue with the HLB cartridge.

### 2.5. Optimization the Method for the Large-Volume Sample 

Although we were able to elute a 0.5 mL sample into the 60 mg HLB cartridge and got a similar recovery (81.4 ± 8.7%), the fluorescence intensity of the 0.5 mL standard, constructed in the sample matrix, was about half (58.1 ± 7.8) of that obtained from eluting 0.25 mL. By eluting 0.5 mL into the 60 mg HLB cartridge, we were able to detect 0.6 ppm OTC in the final extract, which corresponds to 6 µg g^−1^ fresh weight (FWT) in the original sample. On the other hand, we were able to detect 0.3 ppm OTC in the final sample extract, which corresponds to 3 µg g^−1^ FWT by eluting a 1 mL sample or standard (constructed in the sample matrix) into the 500 mg HLB. The OTC recovery (71.8 ± 4.9) obtained using the 500 mg HLB cartridge was similar to that obtained using the 60 mg cartridge.

### 2.6. Application of the New Method and Comparison with the ELISA Kit

**Figure 5:**
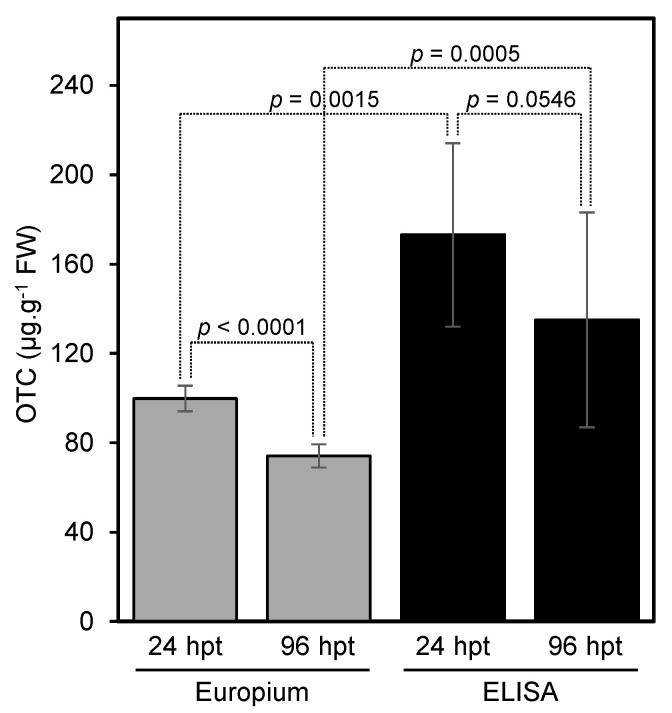
Application of europium method to filed samples and comparison with ELISA kit. OTC was extracted using the HCl and trichloroacetic acid mixture and cleaned using the HLB cartridge before being analyzed using the europium assay. The acidic extract was diluted (1:100) using distilled water, and then (1:4) using the dilution buffer provided by the kit and analyzed directly without any cleanup. Values with *p*-values < 0.05 are significantly different using the two-tailed student *t*-test.

In this section, we used our developed method to measure the level of OTC in leaf samples that were obtained from trunk-injected trees and compare our method with the ELISA method. Our results showed that the new method can be successfully used to determine the level of OTC in trunk-injected citrus trees (Figure 5, Table S1). The levels of OTC 24 and 96 h post-injection as measured by the fluorescence assay were similar to those obtained using the ELISA kit (Figure 5). 

### 2.7. Cross-Reactivity of OTC Metabolites

**Figure 6:**
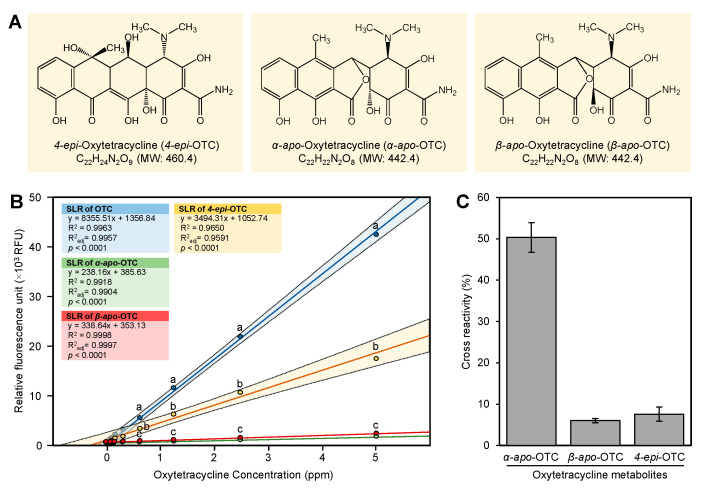
Cross-reactivity of the OTC metabolites with the europium method. (**A**) Chemical structure of OTC metabolites; 4-*epi*-OTC, α-*apo*-OT, β-*apo*-OTC. (**B**) Standard curves 4-epi-OTC, α-apo-OTC, and β-apo-OTC, and OTC as generated using the europium method. (**C**) Cross-reactivity (%) of 4-e*pi*-OTC, α-*apo*-OTC, and β-*apo*-OTC relative to that of OTC. Values with *p*-values < 0.05 are significantly different using two-tailed student *t*-test. Standards (5.0, 2.5, 1.2, 0.6, and 0.3 ppm) were dissolved in water. Assays were performed under the same conditions; 100 µL of standard, 300 µL of tris buffer, 40 µL of EDTA, 40 µL of 1% CTAC, and 20 µL of europium. Responses (relative fluorescence), at the same concentration, with different letters are significantly different using Tukey’s honestly significant difference (HSD) test (*p*-value < 0.05).

In this section, we studied the cross-reactivity of three of the OTC metabolites (4-*epi*-OTC, α-a*po*-OTC, and β-*apo*-OTC) (Figure 6A) with the europium method. The standard curves of OTC, 4-*epi*-OTC, α-*apo*-OTC, and β-*apo*-OTC are shown in Figure 6B. The OTC standard showed the highest fluorescence activity followed by 4-*epi*-OTC (Figure 6B). The α-*apo*-OTC and β-*apo*-OTC showed small cross-reactivity (<7.5%) with the europium fluorescence assay (Figure 6C). On the other hand, the 4-*epi*-OTC showed a high cross-reactivity (50.0 ± 3.6%) with europium fluorescence assay (Figure 6C). 

## 3. Discussion

Plant tissues are complex matrixes, and OTC forms a complex with different cations such as iron and copper. Therefore, the analysis of OTC in plant tissues requires a special procedure. In general, OTC is extracted using an acidic solution that contains a chelating agent such as EDTA or citric acid to compete with the chelating cations and release OTC. Several approaches were employed to remove protein from biological samples and animal tissues including ultrafiltration, heat, the use of strong acids such as HCl and trichloroacetic acid, and the precipitation of proteins using organic solvents acetonitrile [13]. Previous studies also showed that phosphate must be removed from the sample because it reacts with europium and precipitates it [13]. The presence of plant metabolites could also reduce the fluorescence intensity by absorbing the applied and emitted light or by reacting with the reagents of the europium assay. 

The ideal conditions for the formation of a fluorescent OTC:Eu(III) complex occurs over the pH range of 7.7–9.7 [13]. Our preliminary results showed that the fluorescence intensity of the standard was reduced by about five folds when it was prepared in the sample matrix, although the pH was 8.5. This result suggested that the presence of plant metabolites such as phenols and flavonoids could interfere with the fluorescence assay. To test this hypothesis, we studied the effect of phenols and flavonoids on the fluorescence intensity of the OTC–Eu(III) complex. The addition of gallic acid and catechin to the europium assay’s mixture significantly reduced the fluorescence intensity of the OTC standard.

In agreement with our observations, previous studies showed that CTAC reacts with phenols based on an acid–base mechanism [15,16]. CTAC surfactant has been successfully employed for the removal of phenols from water [16]. The reaction of phenols and flavonoids with the CTAC surfactant decreases the amount of available sensitizing agent (CTAC) and consequently decreases the fluorescent intensity. Low fluorescent intensity was observed when CTAC was excluded from the reaction mixture. The formation of the CTAC–phenol complex (yellow color) could also reduce the fluorescence intensity by absorbing the applied or emitted light. Our previous study showed that citrus leaves are rich in phenols and flavonoids [17]. The level of phenols and flavonoids in citrus leaves were 3–15 and 0.2–1 mg g^−1^ fresh weight, respectively [17]. 

We tried to replace CTAC with Triton X-100 to avoid the reaction of phenols with CTAC. Unfortunately, the replacement of CTAC with Triton X-100 significantly reduced the fluorescence intensity of the OTC–Eu(III) complex, indicating that CTAC was a better sensitizing agent than Triton X-100. In agreement with our results, Triton X-100, cetylpyridinium chloride, and sodium dodecyl sulfate did not significantly enhance the fluorescence intensity of the OTC–Eu(III) complex [12]. A previous study also showed that the europium–tetracycline–CTCA system was about six times more sensitive than the europium–tetracycline–Triton system [14]. 

Sample cleanup with the HLB cartridge improved the fluorescence intensity and allowed a better quantification of OTC in spiked samples. In agreement with our results, previous reports showed that quantification of OTC was not feasible without a cleanup step. For example, the OTC residues in rainbow trout were cleaned using a Sep-pak C18 column before HPLC analysis [18]. In another study, OTC extracted from chicken muscles was cleaned using a hydrophilic–lipophilic balanced copolymer (HLB cartridges) before being analyzed using luminescence screening assay [19]. The HLB SPE showed higher recovery and better reproducibility than the typical C18 cartridges [19].

Previous studies showed that methanol was the best eluant for OTC because most of the OTC (≈97%) is eluted from the SPE cartridge in the first mL, and it is compatible with the europium assays [13]. Upon preconditioning of the 60 mg HLB cartridge, OTC was eluted using 1 mL of methanol, as described earlier [13]. However, the spiked sample showed very low fluorescence intensity, indicating that some phenols and flavonoids were eluted with OTC. The highest fluorescence intensity was achieved when the sample extract was cleaned with 2 mL of 20% methanol and OTC was eluted with 1 mL of 60% methanol. The fluorescence intensity of the standard curve that was generated using 4.5% CTCA was about 1.5-fold higher than that built using 1% CTAC, indicating that 4.5% CTAC was better than 1% in the case of sample extract. 

Our results showed that the 60 mg HLB cartridge can be loaded with up to 0.5 mL of the sample extract. However, the fluorescence intensity of the 0.5 mL sample was reduced by a factor of two compared with the 0.25 mL sample. This result indicated that 0.25 mL was the optimum sample volume that can be loaded into the 60 mg HLB cartridge. On the other hand, we were able to load 1 mL of the sample extract into the 500 mg HLB cartridge, which decreased the limit of detection (LOD) to 3 µg g^−1^ FWT. The recovery of OTC from spiked leaf samples ranged from 75 to 89%. Our recoveries are similar to those obtained from spiked muscle (53–63%) and liver (68–78%) tissues and extracted using a mixture of HCl and trichloroacetic acid [18].

The developed method was used to estimate the level of OTC in trunk-injected trees. The OTC level in trunk-injected trees as measured using the developed method was similar to those obtained by the ELISA method, indicating that our method was accurate. The relative standard deviation of the europium assay was also lower than that of the ELISA assay, indicating a high reproducibility for the europium method.

The europium assay showed high cross-reactivity with the main metabolite of OTC, 4-*epi*-OTC (50.0 ± 3.6%), whereas α-a*po*-OTC and β-*apo*-OTC showed very low cross-reactivity (<7.5%). In our previous study, 4-*epi*-OTC also showed high cross-reactivity with the Acell ELISA kit, whereas α-*apo*-OTC and β-*apo*-OTC metabolite did not show any cross-reactivity below 50 ng mL^−1^ [11]. Our current results indicated that 4-*epi*-OTC, which is an epimer of OTC, forms a stable complex with europium. On the other hand, α-*apo*-OTC and β-*apo*-OTC, which are different from OTC, do not bind with europium. The cross-reactivity of europium with the main metabolite of OTC, 4-*epi*-OTC, could be considered as an advantage because OTC and 4-*epi*-OTC in foods are under strict regulation [8].

Eu(III) also forms a complex with tetracycline, chlortetracycline, and doxycycline [14]. The fluorescence intensity of the TC–Eu(III) > OTC–Eu(III) > Chlor-OTC–Eu (III) [14]. Complexation of tetracyclines with europium in the presence of EDTA and CTAC was successfully used to determine the level of tetracyclines in calf serum [14]. Another study also showed that Eu(III) forms a complex with OTC, chlor-OTC, tetracycline, and methacycline in the presence of 1,10-phenanthroline and sodium dodecyl-benzene sulfonate [20]. This fluorometric assay was successfully used to determine the level of tetracycline in blood plasma [20].

OTC has been effectively used to control many plant diseases and was recently approved for the control of Huanglongbing in citrus. Although several methods have been developed for the analysis of OTC, these methods either are not sensitive or time consuming and need expensive instruments. On the other hand, the ELISA assay is considered the most convenient method for the detection of OTC because it is simple, sensitive, (0–50 ppb), fast, and can be used to analyze many samples at the same time. Unfortunately, the ELISA kits are expensive and not available all the time. In addition, long or improper storage of the ELISA kits could result in the loss of their enzymatic activity. Previous studies showed that OTC can be measured using fluorescence or luminescence assay upon complexation with europium. However, this method has not been applied to the plant matrix, which is rich in secondary metabolites. Our results showed that phenols and flavonoids interfere with the europium assay by reacting with CTAC. However, this interference was minimized using an HLB cartridge. Our current results showed that the europium method was sensitive (3 µg g^−1^ FWT), reproducible (RSD; 9.1%), and comparable to the ELISA method. Our results also showed that the developed method could be successfully used to measure the level of OTC in citrus trees upon trunk injection. Since the europium method is sensitive and can detect several tetracyclines, the development of the europium-based method for plant tissues could be a powerful tool for antibiotic research.

## 4. Methods

### 4.1. Preliminary Study

#### 4.1.1. Preparation of OTC Standard

OTC stock solution (200 ppm) was prepared by dissolving 10 mg of OTC in 50 mL of 0.06 M HCL. The stock solution was diluted in water to prepare a series of OTC standards (5, 2.5, 1.25, 0.61, 0.3, and 0.15 ppm) to build the standard curve.

#### 4.1.2. Extraction of OTC

**Figure 7:**
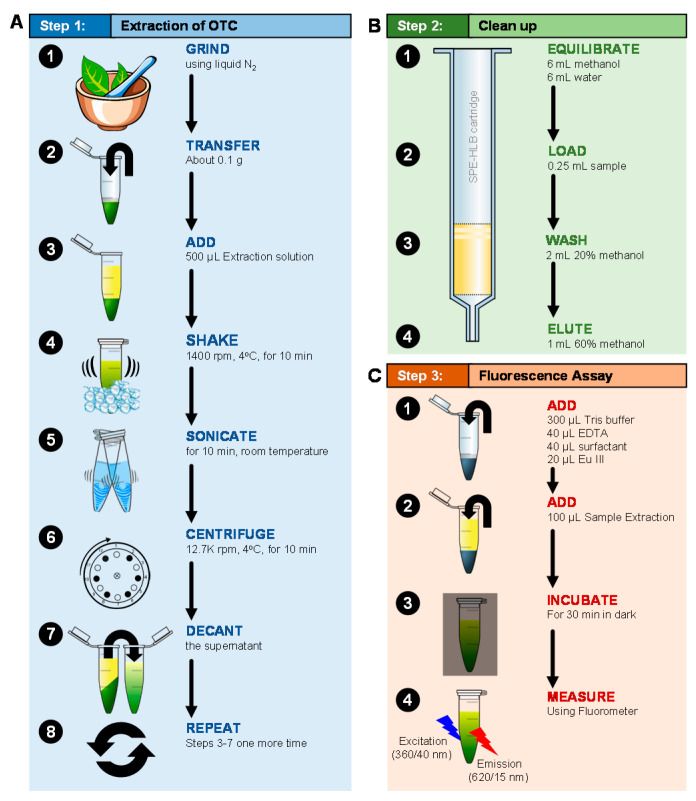
Extraction, cleanup, and europium assay for OTC extracted from citrus leaves. (**A**) Extraction scheme for OTC from ground citrus leaf tissues. (**B**) Cleanup of OTC from citrus leaf samples (0.25 mL) using 60 mg HLB cartridge. (**C**) Fluorescence assay of OTC.

Valencia sweet orange (*Citrus sinensis* (L.) Osbeck) trees were used for the spiking experiment. Trees were 18 months old, and approximately 100 ± 10 cm tall when used. Three leaves were collected from each tree (from the base, middle, and top areas). Collected leaves were ground in liquid nitrogen using a mortar and a pestle (Figure 7A). About 100-mg aliquot of the ground tissues was placed in a 2-mL centrifuge tube and spiked with 50 µL of 200 ppm OTC standard. Control samples were spiked with 50 µL of distilled water. Then, each sample was mixed with 0.5 mL of the extraction solution (0.1 N HCl, 0.01% EDTA, pH adjusted to 4.0 using 1 N NaOH), vortexed for 10 min at 1440 rpm at 4 °C, and sonicated for another 10 min. The samples were centrifuged at 12,700 rpm for 10 min at 4 °C to remove the plant debris (Figure 7A). The extraction procedure was repeated with another 0.5 mL extraction solution, and the supernatant was collected. 

#### 4.1.3. Fluorescence Assay

In our preliminary work, the fluorescence assay was performed without using solid-phase extraction (Figure 7B). A 100-µL aliquot of the standard or sample was mixed with 300 µL tris buffer (0.1 M, pH 8.5), 40 µL EDTA (0.00025 M), 40 µL cetyltrimethylammonium chloride (CTAC) (1%), and 20 µL europium chloride (0.00125 M) (Figure 7C). The mixture was incubated in the dark for 30 min. Then, the fluorescence intensity was measured using a fluorometer (Synergy Multimode reader, Bioteck) (Figure 7C). The excitation wavelength was set to 360 ± 40 nm, the emission wavelength was set to 620 ± 20 nm, and the gain was set to 80. The standard curve was constructed by plotting the OTC concentration versus the relative fluorescence unit (RFU).

#### 4.1.4. Inhibition of Fluorescence by Phenols and Flavonoids 

A100 ppm gallic acid in 5 ppm OTC standard was prepared by mixing 25 µL of the OTC stock solution (200 ppm) with 100 µL of gallic acid (1000 ppm in methanol) and 875 µL methanol. A 1000 ppm gallic acid was prepared by mixing 25 µL of the OTC stock solution (200 ppm) with 200 µL of 5000 ppm gallic acid in methanol, and 785 µL of methanol. Catechin solutions (100 ppm and 975 ppm in 5 ppm OTC) were prepared in the same way using methanol. OTC standard (5 ppm) was prepared by mixing 25 µL of 200 ppm OTC stock solution with 975 µL methanol. The fluorescence assay was performed using 100 µL of each solution and the percentage inhibition of gallic acid and catechin was calculated relative to pure OTC standard, which was considered 100%.

To record the UV-vis spectra for gallic acid– and catechin–CTCA complex, a 100-µL of 100 ppm gallic or catechin was mixed with 40 µL of CTCA, and 300 µL of tris buffer and the UV-visible spectra were measured between 250 and 800 nm using the Gen5 microplate reader (Biotek, Winooski, VT, USA).

#### 4.1.5. Effect of Surfactants Europium on Fluorescence Intensity

The OTC standard curve was repeated as mentioned before without any surfactant, with 40 µL of 1% CTAC, with 40 µL of 4.5% CTAC, and with 40 µL of 10% Triton X-100. The standard that was prepared in the sample matrix and eluted through the 60 mg HLB cartridge was also repeated using 40 µL of 1 and 4.5% of CTAC.

### 4.2. Main Study

#### 4.2.1. Sample Extraction

Citrus leaves were ground in liquid nitrogen using a mortar and a pestle (Figure 7A). About 100-mg aliquot of the ground tissues was placed in a 2-mL centrifuge tube and spiked with 50 µL of OTC standard (200 ppm). Five samples were spiked with OTC standard and another five (controls) were spiked with 50 µL of distilled water. Then, 0.5 mL of the extraction solution (1 M HCl, 2.2% trichloroacetic acid) was added to each sample (Figure 7A) [18]. The second extraction solution was similar to the first solution, but it also contained 0.1% sodium ethylenediaminetetraacetic acid (EDTA). The mixture was vortexed for 10 min at 1400 rpm at 4 °C and then sonicated for another 10 min at room temperature (Figure 7A). To remove the leaf debris, the samples were centrifuged at 12,000 rcf for 5 min at 4 °C (Figure 7A). The extraction procedure was repeated using another 0.5 mL of extraction solution, and the supernatant was collected and stored at –80 °C (Figure 7A). The extraction procedure was repeated in the same way using the second extraction solution. 

#### 4.2.2. Solid-Phase Extraction

##### Oasis 60 mg HLB Cartridge

The Oasis HLB (Waters, Milford, MA, USA) cartridge (3 cc, 60 mg) was preconditioned using 6 mL of methanol and 6 mL of water (Figure 7B). An aliquot of 0.25 mL of the spiked sample was added to the cartridge and was washed with 2 mL of 20% methanol and eluted using 1 mL of 60% methanol (Figure 7B). To determine the level of OTC in spiked samples, a set of standards was prepared in the sample matrix (supernatant from control samples) and cleaned using Oasis HLB (60 mg) as described above (Figure 7B). The fluorescence assay was conducted using 100 µL standard or sample and 40 µL of 1% CTCA and was repeated using 40 µL of 4.5% CTAC as described before (Figure 7C). To improve the detection limit, we also elute 0.5 mL of the spiked sample and standard (standard in sample matrix) into the 60 mg HLB cartridge.

##### HyperSep C18 Cartridge

To compare the efficiency of the HLB cartridge with the C18 cartridge, a 250 µL aliquot of the sample extract was spiked with 25 µL of 200 ppm OTC standard and was cleaned either with the 60 mg HLB cartridge or with a 500 mg (2.8 cc) HyperSep C18 cartridge (Thermo fisher scientific, Waltham, MA, USA). The HLB and C18 cartridge was preconditioned using 6 mL of methanol followed by 6 mL of water. The sample was washed with 2 mL of 20% methanol, and then, OTC was eluted from the HLB or C18 cartridge using 1 mL of 60% methanol. Five spiked samples were eluted from each cartridge. The fluorescence assay was conducted using 100 µL standard or a sample and 40 µL of 1% of CTAC.

##### HLB-500 mg

To increase the sample volume that can be loaded into the HLB cartridge, we repeated the SPE using a 500 mg HLB cartridge. The 500 mg HLB cartridge was preconditioned with methanol (12 mL) and water (12 mL), and then, 1 mL of the sample extract (1 M HCL, 2.2% trichloroacetic acid, 0.1% EDTA) was loaded into the cartridge. The sample was washed with 2 mL of 20% methanol followed by 2 mL of 60% methanol, the OTC was eluted using 2 mL of methanol, and the eluate was concentrated under a gentle nitrogen stream to 1 mL. To determine the level of OTC in spiked samples, a set of standards were prepared in the sample matrix (supernatant from control samples) and cleaned using Oasis HLB (500 mg) as described above. The assay was conducted using 100 µL of the final extract and 40 µL of 4.5% CTAC as described above.

### 4.3. Application of the New Method and Comparison with the ELISA Assay

In this field experiment, we used five-year-old trees of “Hamlin” sweet orange (*Citrus sinensis* (L.) Osbeck) on Swingle citrumelo rootstocks. Five trees were assigned randomly to each treatment. The injection solution was prepared by dissolving 100.0 g (equivalent to 17.3 g of pure OTC) of Fireline 17 WP (Agro Source, Tequesta, FL, USA) in 200 mL of water. Each tree was injected with a 20-mL aliquot of the Fireline solution (equivalent to 1.7 g OTC) using a 20-mL Chemjet manual injector (Healthy Tree PHC, Inc., Norfolk, VA, USA) as described previously [7]. The injector was left overnight until all the solution was taken by the tree. Control plants (5 trees) did not receive any treatment. Injected trees were sampled 24 and 98 h post-injection. Three leaves of each tree were collected from each tree from three different places above the injection site. Leaves collected from different trees were pooled and ground together using liquid nitrogen as described before.

OTC was extracted using the HCl and trichloroacetic acid mixture, cleaned using an HLB cartridge, and analyzed using the fluorescent assay as described before. The ELISA assay was performed using the OTC ACCEL ELISA (Plexense, Inc., Davis, CA, USA). Before performing the ELISA assay, 10 µL of the sample extract was diluted to 1000 µL using distilled water, and then, 50 µL of the diluted sample was further diluted to 200 µL using the dilution buffer provided by the kit.

### 4.4. Cross-Reactivity of OTC Metabolites

OTC, 4-epi-OTC, and β-apo-OTC stock solutions (200 ppm) were prepared in 0.06 M HCl. The stock solution (200 ppm) of α-apo-OTC was prepared in a mixture of methanol and 0.06 M HCl (1:10). A series of dilutions (5, 2.5, 1.25, 0.62, 0.31, and 0.15 ppm) from each standard were prepared in water and used to construct the standard curves.

### 4.5. Statistical Analysis

JMP 9.0 software (SAS, Cary, NC, USA) was used for all statistical analyses. 

The fluorescence intensity of samples eluted through the C18 cartridge was compared to that obtained by the HLB cartridge using a two-tailed student *t*-test (*p*-value < 0.5). The percentage inhibition of gallic acid was compared to that of catechin using a two-tailed student *t*-test (*p*-value < 0.5). The percentage recoveries of OTC obtained using the mixture of HCl and trichloroacetic with and without EDTA were compared to each other using the two-tailed student *t*-test (*p*-value < 0.5). The levels of OTC in trunk-injected trees measured using the europium method and ELISA method were compared to each other also using the two-tailed student *t*-test (*p*-value < 0.5). In the same manner, the level of OTC in trunk-injected trees after 24 h was compared to that after 98 h using the two-tailed student *t*-test (*p*-value < 0.5). Comparison among the mean response (fluorescence) of OTC, 4-*epi*-OTC, and β-*apo*-OTC was performed using Tukey’s honestly significant difference (HSD) test (*p*-value < 0.05). The fluorescence intensity of the OTC standard with and without europium at each concentration was compared to each other using the two-tailed student *t*-test (*p*-value < 0.5). 

## 5. Conclusions

In this study, we showed that the europium-sensitized fluorescence-based method can be successfully used to measure OTC in citrus tissues after a cleanup step using an HLB cartridge. Our results showed that this method was sensitive (3 µg g^−1^ FWT) and reproducible (RSD; 9.1%). We showed that this method can be used to measure the level of OTC in citrus trees upon trunk injection. This method can be used to analyze a large number of samples (96) at the same time using a microplate reader. The europium assay showed high cross-reactivity with the main metabolite of OTC, 4-*epi*-OTC. The high cross-reactivity of 4-*epi*-OTC with the europium method could be an advantage because the level of OTC and 4-*epi*-OTC in foods are under strict regulation. 

## Data Availability

Data is contained within the article.

## Supplementary Materials

The following are available online at https://www.mdpi.com/2079-6382/10/2/224/s1, Table S1: The level of OTC (µg g^−1^ FW) in trunk-injected trees 24 and 96 h post-injection as obtained by the europium and ELISA assay.

## References

[1] Stockwell V.O., Duffy B. (2012). Use of antibiotics in plant agriculture. OIE Rev. Sci. Tech..

[2] McManus P.S., Stockwell V.O., Sundin G.W., Jones A.L. (2002). Antibiotic Use in Plant Agriculture. Annu. Rev. Phytopathol..

[3] Wang N., Pierson E.A., Setubal J.C., Xu J., Levy J.G., Zhang Y., Li J., Rangel L.T., Martins J. (2017). The *Candidatus* Liberibacter–Host Interface: Insights into Pathogenesis Mechanisms and Disease Control. Annu. Rev. Phytopathol..

[4] Blaustein R.A., Lorca G.L., Teplitski M. (2018). Challenges for Managing *Candidatus* Liberibacter spp. (Huanglongbing Disease Pathogen): Current Control Measures and Future Directions. Phytopathology.

[5] Zhang M., Yang C., Powell C.A. (2015). Application of antibiotics for control of citrus huanglongbing. Adv. Antibiot. Antibodies.

[6] 6- Hu J., Wang N. (2016). Evaluation of the Spatiotemporal Dynamics of Oxytetracycline and Its Control Effect Against Citrus Huanglongbing via Trunk Injection. Phytopathology.

[7] Killiny N., Hijaz F., Gonzalez-Blanco P., Jones S.E., Pierre M.O., Vincent C.I. (2020). Effect of Adjuvants on Oxytetracycline Uptake upon Foliar Application in Citrus. Antibiotics.

[8] Le T., Yu H., Zhao Z., Wei W. (2012). Development of a Monoclonal Antibody-Based ELISA for the Detection of Oxytetracycline and 4-*Epi*-Oxytetracycline Residues in Chicken Tissues. Anal. Lett..

[9] Priya S.S., Radha K. (2014). V Brief review of spectrophotometric methods for the detection of tetracycline antibiotics. Int. J. Pharm. Pharmaceeutical Sci..

[10] Al-Rimawi F., Hijaz F., Nehela Y., Batuman O., Killiny N. (2019). Uptake, Translocation, and Stability of Oxytetracycline and Streptomycin in Citrus Plants. Antibiotics.

[11] Hijaz F., Killiny N. (2020). Evaluation of Oxytetracycline Metabolites Cross-Reactivity with Oxytetracycline Enzyme-Linked Immunosorbent Assay (ELISA). Antibiotics.

[12] Jee R.D. (1995). Study of micellar solutions to enhance the europium-sensitized luminescence of tetracyclines. Analyst.

[13] Chen G., Schneider M.J., Darwish A.M., Lehotay S.J., Freeman D.W. (2004). Europium-sensitized luminescence determination of oxytetracycline in catfish muscle. Talanta.

[14] Arnaud N., Georges J. (2001). Sensitive detection of tetracyclines using europium-sensitized fluorescence with EDTA as co-ligand and cetyltrimethylammonium chloride as surfactant. Analyst.

[15] Guo S., Wu Z., Liu W., Huang D., Li H., Hu N. (2016). Enrichment and isolation of phenol from its aqueous solution using foam fractionation. J. Ind. Eng. Chem..

[16] Azmiyawati C., Sawitri E., Siahaan P., Darmawan A., Suyati L. (2020). Preparation of magnetite-silica-cetyltrimethylammonium for phenol removal based on adsolubilization. Open Chem..

[17] Hijaz F., Al-Rimawi F., Manthey J.A., Killiny N. (2020). Phenolics, flavonoids and antioxidant capacities in Citrus species with different degree of tolerance to Huanglongbing. Plant Signal. Behav..

[18] Nordlander I., Johnsson H., Österdahl B. (1987). Oxytetracycline Residues in Rainbow Trout Analysed by a Rapid HPLC Method. Food Addit. Contam..

[19] Schneider M.J., Chen G. (2004). Time-resolved luminescence screening assay for tetracyclines in chicken muscle. Anal. Lett..

[20] Shtykov S.N., Smirnova T.D., Bylinkin Y.G., Zhemerichkin D.A. (2005). Fluorimetric determination of tetracyclines with the europium chelate of 1,10-phenanthroline in micellar solutions of anionic surfactants. J. Anal. Chem..

