# Supplementary material for: Development of Europium-Sensitized Fluorescence-Based Method for Sensitive Detection of Oxytetracycline in Citrus Tissues
Source: Antibiotics (Basel). 2021 Feb 23;10(2):224. doi: 10.3390/antibiotics10020224 (PMC7926362; doi:10.3390/antibiotics10020224)
Supplement: Supplementary file 1 [file antibiotics-10-00224-s001.pdf]

Table S1. The level of OTC (µg g-1 FW) in trunk-injected trees 24 and 96 hr post-injection as obtained by the europium and ELISA assay.

| 24 hr post-injection | |
|---|---|
| ELISA | Europium |
| 157.1 | 95.0 |
| 142.3 | 107.8 |
| 220.0 | 94.2 |
| 96 hr post-injection | |
| ELISA | Europium |
| 160.3 | 75.48 |
| 90.2 | 77.8 |
| 191.5 | 67.0 |
| 101.6 | 80.8 |

OTC was extracted using the HCl and trichloroacetic acid mixture and cleaned using HLB cartridge before being analyzed using the europium assay. The acidic extract was diluted (1:100) using distilled water, and then (1:4) using the dilution buffer provided by the kit and analyzed directly without any cleanup.
